# Nonlinear Associations Between Kidney Function Markers and Body Mass Index Among Women of Reproductive Age in Bangladesh

**DOI:** 10.7759/cureus.84385

**Published:** 2025-05-19

**Authors:** Ferdous Ara, Sneha Sarwar, Tasmim Fahima Ahmad, Nusrat Jahan Shorovi, FNU Asamoni, Md. Ruhul Amin, Sharmili Jahan Prome, Md. Saidul Arefin, Abu Ahmed Shamim, M. Akhtaruzzaman

**Affiliations:** 1 Institute of Nutrition and Food Science, University of Dhaka, Dhaka, BGD; 2 Biotechnology and Bioinformatics, Independent University, Dhaka, BGD; 3 BRAC James P Grant School of Public Health, BRAC University, Dhaka, BGD

**Keywords:** bangladesh, body mass index (bmi), creatinine, kidney function markers, serum urea, uric acid, women of reproductive age (wra)

## Abstract

Background

Normal kidney function is essential for maintaining good health. The objective of the study was to explore the association between socio-demographic characteristics and body mass index (BMI) with serum uric acid, urea, and creatinine levels among women of reproductive age (WRA) in selected areas of Bangladesh.

Methodology

A cross-sectional study was conducted among 178 WRA in selected districts of Bangladesh. Data on demographic, socioeconomic, and anthropometric variables were collected. Blood samples were collected, processed, stored, and analyzed using the DIALAB GmbH semi-automatic biochemistry analyzer DTN-405 (Austria) to measure uric acid, urea, and creatinine.

Results

The study found that 42 women (23.6%) were underweight, while 35 (19.7%) were classified as overweight or obese. The average (mean ± SD) serum levels (mg/dL) were 5.74 ± 2.07 for uric acid, 17.93 ± 9.83 for urea, 0.89 ± 0.28 for creatinine, and 92.18 ± 35.86 mL/min/1.73 m² for estimated glomerular filtration rate (eGFR). Elevated levels of creatinine, uric acid, and urea were observed in 47 (27%), 56 (31.5%), and 73 (41%) participants, respectively. Additionally, chronic kidney disease (CKD) was identified in 32 individuals (18%). Overall, normal kidney function markers were found in 55 participants (about 31%), while 9 participants (about 5%) showed elevated levels for all three markers. The odds of having one or more elevated kidney function markers were significantly higher (adjusted odds ratio (AOR) = 2.8; 95% CI: 1.22-6.42; p < 0.05) among individuals with a BMI greater than 18.5 kg/m² compared to underweight women. After adjusting for related factors, BMI was found to be significantly associated with the risk of elevated kidney function markers.

Conclusion

Only one-third of the WRA displayed normal kidney function indicators, and BMI demonstrated a significant non-linear relationship with one or more elevated kidney function indicators, even within the normal BMI range. Therefore, further studies with larger sample sizes are necessary to gain deeper insights.

## Introduction

The kidney plays a significant role in regulating the body’s acid-base and electrolyte balance. It is also crucial for conserving essential nutrients, modulating hormonal activities, and excreting metabolic end products such as uric acid, urea, and creatinine [1]. Additionally, chronic kidney disease (CKD) is primarily characterized by increased urinary albumin excretion and decreased glomerular filtration rate. It currently affects over 10% of the general population and more than 50% of high-risk groups, including the obese, elderly, and individuals with chronic diseases such as cancer [2]. CKD is a growing public health issue in low- and middle-income countries. For example, the estimated prevalence of kidney disease among the South Asian population was 13.5% in 2022 [3].

Obesity and being overweight are serious public health concerns worldwide. In 2022, the World Health Organization (WHO) reported that 43% of individuals (2.5 billion) aged 18 and above were overweight, with 16% (890 million) suffering from obesity [4]. Obesity can affect the kidneys through the buildup of excess fat in and around the kidneys, which can scar kidney tissues and lead to kidney damage. Being overweight is also significantly associated with the risk of non-communicable diseases [5], including CKD. According to a 2014 study of married women in Bangladesh, the prevalence of underweight, normal weight, and overweight was 24.1%, 46.7%, and 29.2%, respectively [6], underscoring the need to analyze the association between kidney function markers and body mass index (BMI). Previous studies have reported an inconsistent association between BMI and kidney function markers. For instance, a non-linear association between BMI and kidney function markers, such as uric acid, has been reported among Chinese adults [7]. In contrast, another study conducted in China demonstrated a positive linear correlation between serum uric acid and BMI [8].

However, to our knowledge, the association of multiple kidney function markers with BMI among women of reproductive age (WRA) in Bangladesh has not been adequately studied. Therefore, the aim of the study is to determine the associations between socioeconomic status and BMI with serum levels of urea, uric acid, and creatinine in WRA.

## Materials and methods

Study design and population

Data for this analysis were obtained from the Nutrition, Health, and Demographic Survey of Bangladesh 2011 (NHDSBD-2011), a cross-sectional national survey conducted from March 2011 to March 2012. For the NHDSBD study, households were randomly selected from sampling units in Bangladesh's seven administrative divisions. Detailed interviews were conducted using a standardized questionnaire, and blood samples were collected from a sub-sample of women aged 15 to 49 for further examination. The study design has been described in detail elsewhere [9]. The present analysis included 178 women from six districts, distributed across three administrative divisions: Dhaka (29 participants from Narshingdi and Gazipur districts), Chattogram (41 participants from Chattogram district), and Khulna (108 participants from Shatkhira, Kushtia, and Meherpur districts, respectively). Among the participants, 120 were from rural areas and 58 from urban areas.

Socio-demographic and anthropometric data

A standardized and validated questionnaire was used to collect data on socio-demographic characteristics, marital status, household food security, lifestyle, and nutrition [10]. A medical team comprised of paramedics and physicians collected and processed blood samples following established protocols [11]. Anthropometric measurements, such as weight and height, were performed by trained interviewers with graduate-level education using standardized protocols [12]. A precision bathroom scale (Tanita HA-622, Tanita Corporation, Tokyo, Japan) was used to record each participant's weight while they were barefoot. A height scale was used to measure each participant's height to the nearest 0.1 cm.

Blood collection

The process has been described previously [13]. In summary, using disposable syringes, about 6 mL of venous blood was drawn and preserved in simple, dry vacutainer tubes for analysis. The serum samples were then prepared and stored in a freezer at -20 °C. The samples were transported from the collection sites to Dhaka using dry ice in an insulated container.

Biochemical analysis

Serum levels of urea, uric acid, and creatinine were determined by analyzing 178 samples collected from women. Prior to blood collection, participants were required to fast for a minimum of eight hours. Using the semi-automatic biochemistry analyzer DTN-405 from DIALAB GmbH (Austria), the serum levels of creatinine, uric acid, and urea were measured.

To ensure accuracy, every 15th sample was reanalyzed, and 12 samples were analyzed twice to estimate the coefficient of variation (CV).

The CV was calculated using the following formula:

$\text{CV} = {\frac{\text{Standard deviation}}{\text{Mean estimated value of the sample}} \times100}$

From the repeated sample analyses, the average CVs for creatinine, urea, and uric acid were 2.45%, 4.52%, and 3.12%, respectively.

Exposure variables

The demographic and socioeconomic data were presented categorically. Participants were divided into terciles based on their age: ≤23 years, 24 to 28 years, and ≥29 years, to divide them roughly into three equal parts. Participants' educational characteristics were divided into three groups: functionally illiterate (0-5 years of education), secondary incomplete (6-9 years), and secondary complete or above (≥10 years) [14]. Marital status was divided into two categories: married and unmarried. Monthly family income was divided into three categories: ≤ BDT 8,000 (Bangladeshi Taka), BDT 8,001-15,000, and > BDT 15,000. Monthly food expenses were classified as low (up to BDT 6,550), medium (BDT 6,551-9,640), and high (more than BDT 9,640).

BMI was classified according to Asian-specific cutoff points. Based on these standards, individuals were categorized as underweight (<18.5 kg/m²), normal weight (18.5-22.9 kg/m²), and overweight or obese (≥23 kg/m²) [15]. Due to the limited number of overweight participants, overweight and obese individuals were combined into a single category for analysis.

Diagnostic criteria

Normal creatinine level was defined as a serum creatinine level of <1.2 mg/dL [16]. Normal uric acid level was defined as a serum uric acid level of <5.8 mg/dL [17]. Normal urea level was defined as a serum urea level of ≤20 mg/dL [16]. Participants with any one or more values above these levels were classified as having elevated kidney function markers. The estimated glomerular filtration rate (eGFR) was calculated using the Modification of Diet in Renal Disease (MDRD) equation: 

$\text{eGFR} = 186.3 \times (\text{Creatinine})^{-1.154} \times (\text{Age})^{-0.203}$

with an additional multiplication factor of 0.742 for females [18]. CKD was defined as eGFR < 60 mL/min/1.73 m², while non-CKD was classified as eGFR ≥ 60 mL/min/1.73 m² [19].

Statistical analysis

All statistical analyses were performed using IBM SPSS Statistics for Windows, Version 26 (Released 2019; IBM Corp., Armonk, New York) and STATA version 16.0 (StataCorp LLC, College Station, Texas). Descriptive analyses were conducted for all variables. The chi-square test assessed associations between socio-demographic factors and kidney health indicators (serum urea, uric acid, and creatinine). Binary logistic regression identified determinants of elevated kidney function markers and eGFR. A smooth curve was generated in STATA to visualize trends between BMI, kidney function markers, and eGFR.

Ethical approval

The study received approval from the Ethical Review Committee of the Faculty of Biological Sciences, University of Dhaka (Ref. No. 10/Biol. Scs./2011-2012). Written informed consent was obtained from all participants prior to data and sample collection.

## Results

Table 1 presents the basic characteristics of the study population (n = 178). Among the participants, 64 women (36%) were aged between 24 and 28 years, with a median age of 25 years. A total of 171 women (96.1%) were married, and 163 (91.6%) were housewives. Nearly half of the participants (87, 48.9%) had not completed secondary education. The median monthly income and food expenditure were BDT 10,000 and BDT 7,541.50, respectively. The median BMI of the participants was 20.54 kg/m². According to Asian cutoffs, approximately 35 women (19.7%) were classified as overweight. 

**Table 1:** Basic demographic characteristics of the study population (N=178). BDT, Bangladeshi Taka; SD, standard deviation; IQR, interquartile range.

Variable	n (%)
Age (years)	
≤23	65 (36.5)
24-28	64 (36.0)
≥29	49 (27.5)
Mean ± SD	25.69 ± 5.66
Median (IQR)	25.00 (22.00, 29.00)
Range	13-50
Marital status	
Unmarried	7 (3.9)
Married	171 (96.1)
Occupation	
Housewife	163 (91.6)
Other	15 (8.4)
Residence	
Rural	120 (67.4)
Urban	58 (32.6)
Education level	
Functionally illiterate (0–5 years)	64 (36.0)
Secondary incomplete (6–9 years)	87 (48.9)
Secondary complete or above (≥10 years)	27 (15.2)
Monthly food expenditure (BDT)	
≤6,550.00	58 (32.6)
6,551.00 – 9,640.00	62 (34.8)
≥9,641.00	58 (32.6)
Mean ± SD	10,076.60 ± 8,817.21
Median (IQR)	7,541.50 (5,900, 11,525)
Range	2,750-90,000
Monthly family income (BDT)	
≤8,000	69 (38.8)
8,001–15,000	70 (39.3)
≥15,001	39 (21.9)
Mean ± SD	13,979.15 ± 12,501.84
Median (IQR)	10,000.00 (7,000.00, 15,000.00)
Range	2,500- 80,000
Body mass index (BMI, kg/m²)	
Underweight (<18.5)	42 (23.6)
Normal (18.5–22.9)	101 (56.7)
Overweight or obese (≥23.0)	35 (19.7)
Mean ± SD	21.15 ± 3.44
Median (IQR)	20.54 (18.64, 22.53)
Range	15.96-32.30

Overall status of all three kidney function markers

Table 2 presents biochemical parameters, including serum creatinine, uric acid, and urea levels among the participants. The median serum levels of creatinine, uric acid, and urea were 0.88 mg/dL, 5.41 mg/dL, and 15.76 mg/dL, respectively. The median estimated glomerular filtration rate (eGFR) among the participants was 84.57 mL/min/1.73 m².

**Table 2:** Biochemical parameters of kidney function marker of WRA. SD, standard deviation; IQR, interquartile range; eGFR, estimated glomerular filtration rate; WRA, women of reproductive age.

Variable	Mean ± SD	Median (IQR)	Range
Creatinine (mg/dL)	0.89 ± 0.28	0.88 (0.72, 1.00)	0.28-1.86
Uric acid (mg/dL)	5.75 ± 2.07	5.41 (4.56, 6.21)	2.42, 12.97
Urea (mg/dL)	17.93 ± 9.83	15.76 (11.79, 19.86)	4.47, 50.14
eGFR (mL/min/1.73 m²)	92.18 ± 35.86	84.57 (71.78, 106.48)	33.86, 310.00

**Figure 1:**
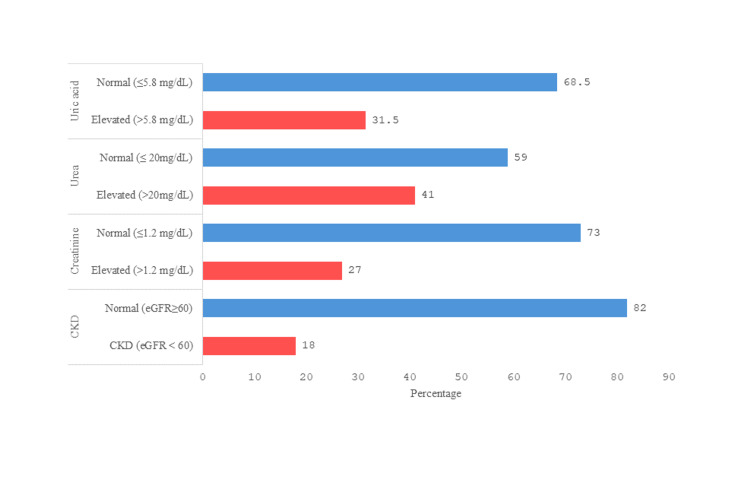
Percentage of participants with normal and elevated kidney function markers and CKD. Elevated values were defined as follows: uric acid >5.8 mg/dL, urea >20 mg/dL, and creatinine ≥1.2 mg/dL. CKD was defined as eGFR <60 mL/min/1.73 m², and non-CKD as eGFR ≥60 mL/min/1.73 m². CKD, chronic kidney disease; eGFR, estimated glomerular filtration rate.

Figure 1 illustrates that elevated serum uric acid, urea, and creatinine levels were observed in 56 (31.5%), 73 (41%), and 48 (27%) participants, respectively. Additionally, CKD was present in 32 (18%) of the participants.

**Figure 2:**
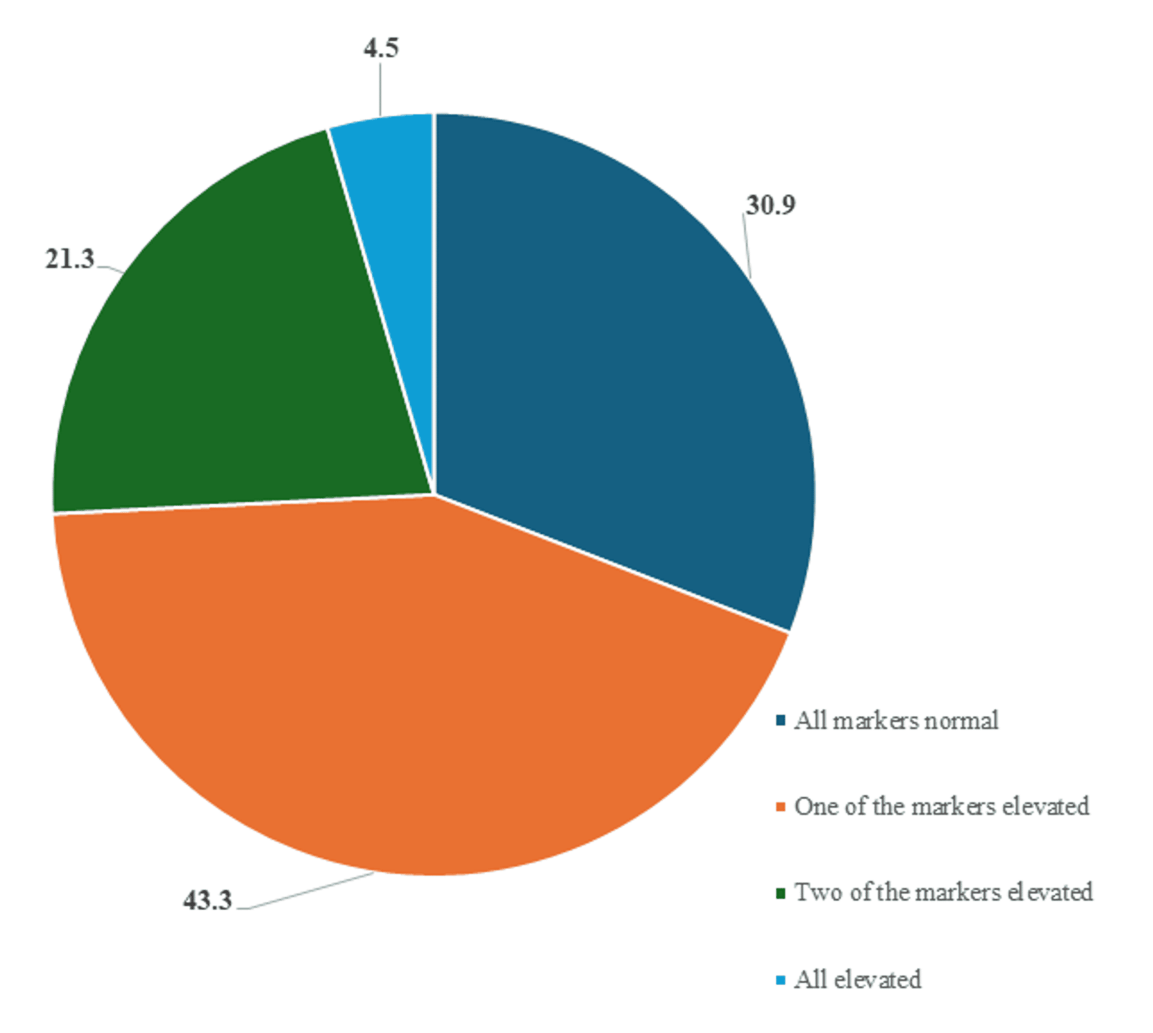
Prevalence of elevated kidney function markers.

Figure 2 shows that when the three parameters (serum urea, uric acid, and creatinine) were considered together, only about 55 women (31%) had all three within the normal range. In contrast, approximately 77 participants (43%) had at least one elevated marker, while around 9 women (5%) had all three markers elevated.

Factors affecting elevated kidney function

As presented in Table 3, individuals with one or more elevated kidney function markers were predominantly aged between 24 and 28 years (49, 39.8%), had incomplete secondary education (66, 53.7%), and maintained a normal BMI (74, 60.2%). A total of 50 individuals (40.7%) reported a monthly household income between BDT 8,001 and 15,000, while 46 individuals (37.4%) had food expenditures ranging from BDT 6,551 to 9,640. 

**Table 3:** Association of different factors with elevated kidney function makers among WRA *p *< 0.05 was considered statistically significant. BDT, Bangladeshi Taka.

Variable	All three normal markers (n=55)	One or more elevated markers (n=123)	*p*-value
				Age (years)			
≤23	21 (38.2)	44 (35.8)	0.207
24-28	15 (27.3)	49 (39.8)	
≥29	19 (34.5)	30 (24.4)	
Occupation			
Housewife	49 (89.1)	114 (92.7)	0.425
Other	6 (10.9)	9 (7.3)	
Residence			
Rural	35 (63.6)	85 (69.1)	0.472
Urban	20 (36.4)	38 (30.9)	
Education level			
Functionally illiterate (0–5 years)	26 (47.3)	38 (30.9)	
Secondary incomplete (6–9 years)	21 (38.2)	66 (53.7)	0.094
Secondary complete or above (≥10 years)	8 (14.5)	19 (15.4)	
Monthly family income (BDT)			
≤8,000	27 (49.1)	42 (34.1)	0.114
8,001–15,000	20 (36.4)	50 (40.7)	
≥15,001	8 (14.5)	31 (25.2)	
Monthly food expenditure (BDT)			
≤6,550.00	24 (43.6)	34 (27.6)	0.109
6,551.00–9,640.00	16 (29.1)	46 (37.4)	
≥9,641.00	15 (27.3)	43 (35.0)	
Body mass index (BMI, kg/m²)			
Underweight (<18.5)	19 (34.5)	23 (18.7)	0.07
Normal (18.5–22.9)	27 (49.1)	74 (60.2)	
Overweight or obese (≥23.0)	9 (16.4)	26 (21.1)	

In the logistic regression analysis, BMI (18.5-22.9 kg/m²) was significantly associated with an increased risk of having at least one elevated kidney function marker among women after adjusting for age, monthly expenditure, and education. The odds of having one or more elevated kidney function markers were significantly (p < 0.05) 2.8 times higher when the BMI was greater than 18.5 kg/m² (Table 4). However, none of the socio-demographic factors were significantly associated with CKD. No significant association between BMI and CKD (calculated from eGFR) was observed in our study (data not shown). 

**Table 4:** Factors associated with the prevalence of at least one elevated kidney function makers (serum creatinine, urea, and uric acid levels). *p-value < 0.05. BDT, Bangladeshi Taka; COR, crude odds ratio; AOR, adjusted odds ratio.

Variable	COR (95% CI)	*p*-value	AOR (95% CI)	*p*-value
Age (Years)				
≤23	1		1	
24-28	1.56 (0.72, 3.39)	0.263	1.25 (0.53, 2.93)	0.607
≥29	0.75 (0.35, 1.64)	0.474	0.56 (0.23, 1.36)	0.199
Education level				
Functionally illiterate (0–5 years)	1		1	
Secondary incomplete (6–9 years)	2.15 (1.07,4.33)	0.032*	2.08 (0.98, 4.44)	0.057
Secondary complete or above (≥10 years)	1.63 (0.62, 4.27)	0.324	1.3 (0.45, 3.74)	0.63
Monthly food expenditure (BDT)				
≤6,550.00	1		1	
6,551.00–9,640.00	2.03 (0.94, 4.39)	0.073	1.84 (0.80, 4.21)	0.152
≥9,641.00	2.02 (0.92, 4.44)	0.079	1.92 (0.793, 4.66)	0.148
Body mass index (BMI, kg/m²)				
Underweight (<18.5)	1		1	
Normal (18.5–22.9)	2.26 (1.07, 4.8)	0.033*	2.8 (1.22, 6.42)	0.015*
Overweight or obese (≥23.0)	2.39 (0.90, 6.31)	0.079	2.29 (0.77, 6.8)	0.134

**Figure 3:**
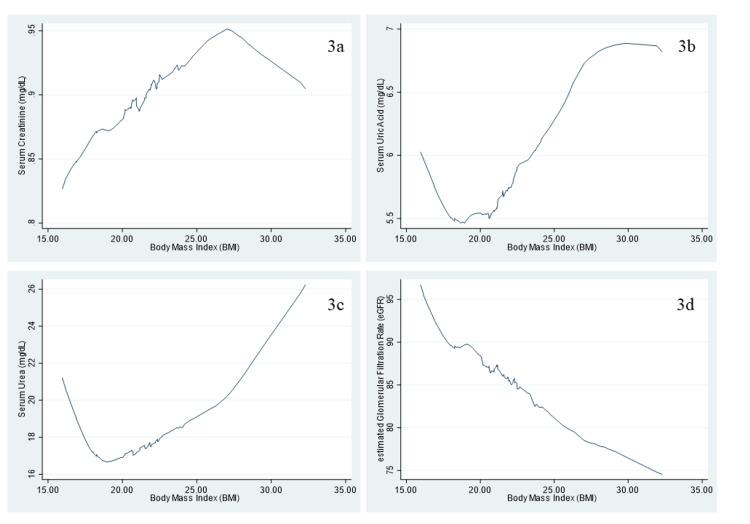
Smooth curves illustrate the association between body mass index (BMI, kg/m²) and kidney function parameters. (3a) Creatinine (mg/dL), (3b) uric acid (mg/dL), (3c) urea (mg/dL), and (3d) estimated glomerular filtration rate (eGFR, mL/min/1.73 m²).

Figure 3 illustrates the non-linear association between BMI and kidney function markers. For creatinine, there is a positive relationship with BMI up to approximately 27.05 kg/m², after which the association becomes negative as BMI continues to increase. In the case of uric acid, a negative association is observed when BMI is around ≤20.6 kg/m², but as BMI rises, uric acid levels also increase. Similarly, for urea, a negative association is seen when BMI is approximately ≤19.0 kg/m², followed by an increase in urea levels as BMI increases. The smooth curve between eGFR and BMI shows a negative association, indicating that eGFR tends to decline with increasing BMI.

## Discussion

The aim of this study was to determine the association of socioeconomic conditions with serum urea, uric acid, and creatinine levels among WRA in selected areas of Bangladesh. Our findings revealed that only about one-third of the participants had all kidney function markers within the normal range, while approximately 80% of them had a normal eGFR of ≥60 mL/min/1.73 m². In the logistic regression analysis, a BMI within 18.5-24.9 kg/m² was found to be significantly (*p* < 0.05) associated with approximately 2.8 times higher risk of having at least one elevated kidney function marker.

The present study shows that the average levels of serum creatinine, urea, and uric acid are similar to findings from studies in other countries. For instance, in a study from northern Ethiopia, the average (mg/dL) values of serum uric acid, urea, and creatinine were 4.43 ± 0.15, 5.67 ± 2.12, and 0.50 ± 0.01, respectively [20]. Furthermore, the mean values of serum uric acid, urea, and creatinine were 3.86 ± 0.92, 26.46 ± 3.55, and 0.58 ± 0.283 among normotensive women in Chennai, India [21]. The slight differences observed may be attributed to variations in study populations, as those studies focused on pregnant women.

In our study, around two-thirds of the participants had one or more elevated kidney function parameters. An elevated level of any kidney function marker is associated with various factors. Fluctuations in serum urea levels are typically caused by protein intake, endogenous protein catabolism, hydration status, hepatic urea synthesis, and renal urea excretion [16]. Elevated urea levels affect organ function and the course of chronic renal disease by causing carboxylation, insulin resistance, reactive oxygen species (ROS) generation, and inflammation [22]. Elevated serum uric acid, or hyperuricemia, also reflects accelerated purine degradation in high cell turnover states and reduced excretion from the kidneys. Hyperuricemia can cause gout and is highly associated with acute kidney injury [23,24].

Our study found that women with a higher BMI (18.5-22.9 kg/m²) had a significantly higher risk of having one or more elevated kidney function parameters than women with a lower BMI (<18.5 kg/m²). The alteration of kidney function markers with BMI also aligns with existing literature, which demonstrates a positive association between BMI and serum urea, uric acid, and creatinine levels [25,26]. One previous study reported that serum uric acid was considerably higher in obese individuals than in underweight individuals, and this elevation increased linearly with BMI, which is approximately 2.98 times greater in overweight people and 5.96 times greater in obese people than in underweight people [8].

In terms of the association between BMI and individual kidney function markers, we observed two different scenarios. According to our findings, BMI appeared to be non-linearly associated with serum urea and uric acid. Such J-shaped relationships were previously reported among females in terms of BMI and uric acid [7]. A possible reason for this is that BMI can lead to elevated mRNA expression and activity of xanthine oxidoreductase, a key enzyme involved in serum uric acid production, as well as increased uric acid secretion. BMI was positively associated with creatinine (up to around 27.0 kg/m²), after which BMI appeared to be inversely associated with creatinine. The inverse association between higher BMI and creatinine levels may be due to the fact that creatinine synthesis is primarily influenced by muscle mass, not fat. Since higher BMI is often associated with increased adiposity rather than muscle mass, this may explain the observed lower creatinine levels in individuals with higher BMI [12].

In terms of eGFR, as BMI increased, the eGFR rate was observed to decline. Similar findings have been reported in overweight or obese individuals, where altered body composition, particularly reduced muscle mass, affects both creatinine levels and eGFR [27].

According to the World Health Organization (WHO), a BMI ≥25 kg/m² is classified as overweight. However, for South Asian populations, a lower threshold of BMI ≥23 kg/m² is recommended due to their higher risk of metabolic complications at comparatively lower BMI levels [28]. Interestingly, a previous study suggests that Bangladeshi women tend to accumulate excess adipose tissue (up to 30%) at substantially lower BMI values (around 21 kg/m²) [29]. In alignment with this, our study also found that women with a normal BMI between 18.5 and 22.5 kg/m² exhibited altered kidney function markers. The accumulation of excess adipose tissue at lower BMI ranges in Bangladeshi women may contribute to the presence of altered kidney function markers.

Evidence from this study highlights that standard BMI thresholds may not fully capture the risk, especially given the unique body composition characteristics of Bangladeshi WRA. Public health initiatives should prioritize the promotion of balanced diets, physical activity, and supportive environments that facilitate healthy lifestyle choices among WRA. Implementing targeted CKD screening programs that include women even within the normal BMI range will be vital in mitigating renal risks in this population. Existing screening policies should incorporate region-specific adiposity measures to improve early detection and preventive strategies.

Strengths and limitations

This study provides valuable insights into the association between kidney function markers (serum urea, uric acid, and creatinine) and BMI, as well as socioeconomic conditions, among WRA in Bangladesh. One of the major strengths lies in the use of standardized laboratory methods for biomarker analysis, along with repeated quality control assessments to ensure data accuracy and reliability.

However, the cross-sectional design of the study limits the ability to establish causal relationships. Furthermore, important confounding factors such as dietary intake, hydration status, and physical activity were not included in the analysis. Although adjustments were made for several variables, including age, education, and monthly food expenditure, other unmeasured factors may also influence kidney function markers and should be considered in future research. Additionally, future research should explore the association of kidney function markers with other anthropometric measures, including waist circumference and body fat percentage.

## Conclusions

The study found that only one-third of WRA in selected regions of Bangladesh had all kidney function markers within the normal range. Notably, BMI showed a significant non-linear association with elevated kidney function markers, even within the traditionally defined normal range. These findings suggest that standard BMI classifications may underestimate renal risk in South Asian populations and underscore the need for tailored risk assessment strategies.

Given the cross-sectional nature of the study and the absence of certain confounding variables, further longitudinal studies with larger, nationally representative samples are needed to confirm these associations and enhance understanding of kidney health among Bangladeshi women.
